# Tuberculosis infectious pool and associated factors in East Gojjam Zone, Northwest Ethiopia

**DOI:** 10.1186/s12890-019-0995-3

**Published:** 2019-11-29

**Authors:** Mulusew Andualem Asemahagn, Getu Degu Alene, Solomon Abebe Yimer

**Affiliations:** 1 School of Public Health, College of Medicine and Health Sciences, Bahir Dar University, Bahir Dar, Ethiopia; 2 Department of Microbiology, Unit for Genome Dynamics, Faculty of Medicine, University of Oslo, Oslo, Norway; 3 Coalition for Epidemic Preparedness Innovations (CEPI), Oslo, Norway

**Keywords:** TB management time, TB infectious pool, Factors, East Gojjam zone, Ethiopia

## Abstract

**Background:**

Globally, tuberculosis (TB) lasts a major public health concern. Using feasible strategies to estimate TB infectious periods is crucial. The aim of this study was to determine the magnitude of TB infectious period and associated factors in East Gojjam zone.

**Methods:**

An institution-based prospective study was conducted among 348 pulmonary TB (PTB) cases between December 2017 and December 2018. TB cases were recruited from all health facilities located in Hulet Eju Enesie, Enebse Sarmider, Debay Tilatgen, Dejen, Debre-Markos town administration, and Machakel districts. Data were collected through an exit interview using a structured questionnaire and analyzed by IBM SPSS version25. The TB infectious period of each patient category was determined using the *TB management time* and sputum smear conversion time. The sum of the infectious period of each patient category gave the infectious pool of the study area. A multivariable logistic regression analysis was used to identify factors associated with the magnitude of TB infectious period.

**Results:**

Of the total participated PTB cases, 209(60%) were male, 226(65%) aged < 30 years, 205(59%) were from the rural settings, and 77 (22%) had comorbidities. The magnitude of the TB infectious pool in the study area was 78,031 infectious person-days. The undiagnosed TB cases (44,895 days), smear-positive (14,625 days) and smear-negative (12,995 days) were major contributors to the infectious pool. The overall average median *TB management time* was 142.4 days (IQR, 98–238 days). Similarly, the average sputum smear conversion time of PTB cases (new and repeat) was 46 days. Residence, knowledge, form of TB, smoking, alcohol history, distance from the facility, comorbidity history and stigma were statistically significant factors TB infectious period (*p*-value< 0.05).

**Conclusions:**

The magnitude of the TB infectious pool is high even if it is lower than the findings of previous studies. This might be an indicator of poor access to TB services, service delays, low community awareness, impaired facility readiness, and poor transportation. Improving personal awareness and behavior, timely management of commodities, and using the *TB management time* in TB control are crucial to improving TB control activities.

## Background

Tuberculosis (TB), one of the world’s deadliest infectious diseases, is mostly caused by *Mycobacterium tuberculosis (*MTB*)* [1, 2]. Although millions of lives have been saved as a result of effective anti- TB interventions, TB is still a major global public health threat with the highest-burden in resource-poor countries [3, 4]. Globally, over 10 million new cases and 1.6 million total deaths occurred as a result of TB in 2017 [2].

Sub-Saharan Africa contributed about a quarter of the global TB burden. Fourteen of the 30 high TB burden countries were from Africa [2]. Similarly, recent evidence indicated that TB is a primary public health agenda in Ethiopia with varying prevalence across regions and districts [5–10]. The emergence of multidrug-resistant TB (MDR-TB) [11–13], limited diagnostics [6–8, 10, 12], low TB case detection and monitoring [14, 15], poor data quality [6, 14, 15], high prevalence of comorbidities [10, 16–19], poor socioeconomic status [19–21], personal behavior [6, 22], and poor service quality [2, 23–26] are challenges of TB control programs. These may contribute to increased TB incidence and lengthy infectious period, which ultimately results in disease complications and high TB transmission [7, 14, 15, 27, 28].

Estimating burdens from TB and monitor the activities of TB control programs is vital to scale up the achievements of TB control programs worldwide. Currently, the world health organization is using case notification (CN), periodic national TB survey and data audit to estimate the burden and monitor TB control programs. Although these strategies are contributing more in TB control activities, they have some feasibility and quality-related limitations in developing countries where poor data quality (inaccuracy, incompleteness and none timeliness) [7, 14, 15, 29], and scarcity of budget [7, 14, 15] are key problems. Therefore, estimating the total number of days that active PTB patients from all categories stay infectious could be important to complement the existing WHO strategies [14, 15].

With this in mind, two studies were conducted in Ethiopia in 2009 [15] and 2014 [14] and introduced a new concept, “*TB management time*”, time interval from the onset of cough to the initiation of treatment, to measure the size of the TB infectious pool and evaluate TB control activities at local levels. Those former studies recommended the applicability of the new approach as an alternative tool after reassessing and improving its limitations. Among the limitations were 1). Infectious period estimations for the different categories of TB patients were based on data obtained from patients who only attended public health facilities, 2). Data used for estimating the sputum smear/culture conversion time to estimate the infectious period after treatment initiation was not based on locally available data, 3). The former studies did not identify factors associated with the total TB infectious period. Therefore, by addressing these limitations, the current study aimed at applying TB management time to measure and analyze factors associated with the total infectious period in East Gojjam Zone of the Amhara Region, Ethiopia.

## Methods

### Study design and setting

A facility-based prospective study was conducted between December 2017 and December 2018 among 348 adult PTB patients on treatment. The study was conducted in East Gojjam Zone, one of the eleven zones of the Amhara Region, Ethiopia. The East Gojjam zone has a total estimated population of 2,632,632 (2,237,737 rural and 394,895 urban). The zone has about 14,010 km^2^ area coverage divided into 18 administrative districts. The zone has about 517 public (406 health posts, 102 health centers, and 9 hospitals) and 100 private health facilities, but only 120 (9 hospitals, 102 health centers, 9 private clinics) were eligible to provide TB diagnostic and treatment services during the beginning of study period [19, 30]. Considering the rule of thumb, 30% of the districts; Hulet Eju Enesie, Enebse Sar Mider, Dubay Telatgen, Dejen, Debre-Markos town administration, and Machakel districts were randomly selected as study sites. All the 38 health facilities in these districts: 2 hospitals, 32 health centers, and 4 private clinics were included.

### Source and study population

All the newly diagnosed smear-positive, smear-negative PTB cases, and retreatment cases taking anti-TB treatment were source population. Whereas, all the adult PTB cases (aged ≥15 years) attending anti-TB in the selected districts were the study population. TB cases who were seriously ill and unable to understand and respond to questions were excluded.

### Sample size determination and sampling techniques

The sample size (348) was calculated by Epi Info version7 using 95% confidence interval, 5% margin of error, 71% average estimated PTB infectious period from the previous studies [14, 15], and 10% non-response rate. To select study sites, we considered the rule of thumb and included 30% of 18 districts through a lottery method. Then all the health facilities in those selected districts (32 health centers, 2 hospitals, and 4 private clinics) were study facilities. All the adult PTB patients taking treatment in those facilities were considered as study participants until getting our sample size.

### Variables and measurement

**Dependent variable**: the magnitude of TB infectious period (number of total infectious days per 100,000 people, High/Low).

In this study, the outcome variable was measured in two ways; the number of total infectious days from all PTB patient categories per 100,000 people, and High/Low based on the median infectious period. The median from the total infectious periods was 125 days and used as a cutoff value as follows; infectious period above the median score (125 days) was considered as high (codded by one) and low (codded by zero) if it was 125 days and below.

### Independent variables

**Socio-demographic variables**: age, sex, religion, education level, residence, marital status, occupation, family size, monthly income, and distance from the facility.

**Clinical profile variables**: a form of TB, category of TB, comorbidities, cough presence, HIV status, and presence of cough with sputum.

**Personal behavior variables**: smoking, chat chewing, alcohol history, the first action to TB, and knowledge on TB.

**Environmental variables**: stigma, contact history, and type of health facility visited.

### Definition of variables

The national comprehensive tuberculosis, leprosy, and TB/HIV training manual for health care workers was used to diagnose, classify and define TB cases [31].

**New TB case**: a patient who has never taken anti-TB treatment/taken for less than one month.

**Smear-positive PTB**: a person who has at least one positive result on AFB microscopy; or whose Expert MTB/RIF test result detected Mycobacterium with susceptibility to rifampicin.

**Smear-negative PTB**: a person who has two negative results on AFB microscopy; and Expert MTB/RIF test detects on Mycobacterium and empirical decision to treat with a full course of anti-TB regimen is made with evidence from supporting tests and clinical decision.

**Retreatment cases**: are patients who have received one month or more of anti-TB drugs in the past, which includes treatment default, failure, and relapse cases. A default case is a patient who was previously confirmed as a defaulter and came back for anti-TB treatment. Treatment after failure is a patient who was confirmed as a failure (smear-positive results after fifth-month treatment) and came back for retreatment. A relapse case is a patient who was previously declared cured or treatment completed and is currently diagnosed with bacteriologically positive (sputum smear or culture), either true relapse or new infection.

**TB infectious pool**: is the total number of days that active TB patients from all categories stay infectious.

**Good knowledge**: if a TB patient answered above the mean score of 12 knowledge questions.

**TB management time** is the time interval from the onset of cough to the anti-TB treatment initiation. It was estimated using the median time interval for each TB category.

**Smoking**: if a TB patient has a history of cigarette smoking of any type, dose, and frequency.

**Alcohol use**: if a TB patient has a history of taking any type of alcohol as per the WHO standard to measure the frequency of alcohol drinking.

**Chat chewing**: if a TB patient has chewed chat with any type, dose, and frequency.

**Smear conversion time**: a time when a PTB case on anti-TB drugs became smear-negative.

**Informal services**: services from traditional healers, spiritual places, and illegal drug sellers.

### Data collection tools and techniques

Data were collected using a semi-structured questionnaire adapted from the previous studies (15, 16). The questionnaire consisted of questions related to sociodemographic, comorbidity, clinical profile, behavior, knowledge, TB symptoms, and health-seeking practices. It was first developed in English, translated to the local language (Amharic), and back to English to check its consistency. The questionnaire was pre-tested among the non-sampled PTB cases to check its clarity and consistency. Six data collectors (nurses and health officers), and three public health practitioners with MPH (supervisors) who took training for three days collected data through an exit interview based on the data collection guideline book. Data collectors also reviewed TB and laboratory registrations for complementing and cross-checking data.

### Data quality assurance

Investigators validated and pre-tested the questionnaire before data collection. A user-friendly data collection guidebook was developed and used during data collection. Data collectors and supervisors took training on the questionnaire, data collection guide book, and data collection procedures for three days. Supervisors and investigators did regular supportive supervision. In addition to checking data completeness and consistency, data collectors and supervisors cross-checked data of each participant with TB logbooks daily.

### Data processing and analysis

In this study, both descriptive and analytical statistics were computed using IBM SPSS version 25. Proportions, mean, median and interquartile range were some of the descriptive statistics to estimate TB management time and TB infectious pool. The TB management time, the time interval from the onset of cough to the initiation of anti-TB treatment, for each PTB patient category (new smear-positive, new smear-negative, retreatment, and undiagnosed cases) were estimated using median statistics.

The median *TB management time* for new smear-positive PTB patients was computed based on data collected from smear-positive PTB patients. Besides TB management time, PTB cases remain infectious for some time period after the treatment initiation [11, 14, 32–34]. Taking this into account, we determined the sputum smear conversion time using sputum smear microscopy. Smear positive PTB patients were followed up weekly through the sputum smear test for up to 22 weeks until they underwent smear conversion. To estimate the total infectious period from the onset of cough to time of non-infectiousness, a median infectious period after treatment initiation was added to the time management of smear-positive cases.

Similarly, the TB management time for the smear-negative PTB cases was calculated based on data from smear-negative TB cases with similar procedures applied for smear-positive TB cases. In this case, the number of smear-negative culture-positive infectious TB cases was estimated using a national 57% smear-negative culture-positive proportion since no sputum culture service was given in the study area [12, 14]. The total infectious period of new smear-negative PTB patients was estimated by multiplying the TB management time with the calculated number of smear-negative culture-positive TB cases.

Also, the median TB management time was calculated for each re-treatment category; relapse, failure, and default cases. To compute the median TB management time of relapse cases, we considered two-time intervals; the time interval between the first onset of cough to the first treatment initiation and the time interval between the reoccurrence of cough to the start of re-treatment. Similarly, the time interval between the first onset of cough to the date of failure was a TB management time for treatment failure cases. Also, the time period from the initial start of the cough to the start of retreatment was taken as TB management time for the defaulted cases. The infectious period after retreatment initiation was estimated based on the sputum culture conversion time of MDR-TB cases. In Ethiopia, sputum culture conversion time among MDR-TB cases varied from 30 to 72 days [11, 14, 34]. We added an average of 56 days to the TB management time of each retreatment case to estimate its infectious period.

For estimating the magnitude of the TB infectious pool of the study area, the contribution of undiagnosed TB cases was also considered. The number of undetected TB cases was estimated based on the 2018 East Gojjam Zone Health Department report where the undetected PTB cases accounted for about 51% of the detected PTB cases [19]. Based on the literature, undiagnosed TB cases were infectious for three years, but we used 365 days since our aim was to estimate the annual infectious period [14]. Thus, TB management time of the undiagnosed TB cases was calculated by multiplying the number of calculated undetected PTB cases with 365 days.

**Fig. 1:**
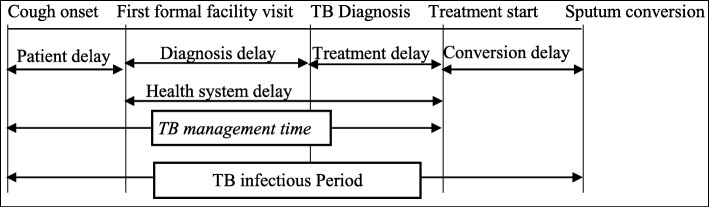
A revised model describing the total TB infectious period in Ethiopia, 2018

Then the total infectious periods of PTB patients (smear-positive, smear-negative, retreatment and undiagnosed TB cases) were calculated by multiplying the total infectious periods for each patient category with the total number of cases in each category (Fig. 1). Lastly, the infectious pool of the study area was estimated using the sum of infectious periods of each PTB patients using the following equation. Total infectious pool = A1Ni + … + A7N7+ [14, 15], where A1 and N1, A2 and N2, A3 and N3, A4 and N4, A5 and N5, A6 and N6 and A7 with N7 are the median infectious period and total number for smear- positives, smear-negatives, relapse, treatment failures, default cases, and undiagnosed cases, respectively.

In addition, bivariate and multivariable logistic regression analyses were used to identify factors associated with the magnitude of TB infectious period in the study area. Variables with a *p*-value of 0.2 at the bivariate logistic regression analysis were used to select candidate variables to fit the multivariable logistic regression model. The outcome variable was labeled high, which was coded as one, and low, which was coded as zero, based on the descriptions stated on the variable and measurement section. A stepwise forward LR method was used for data analysis. The goodness of fit test was checked using the Hosmer-Lemeshow test and the receiver operating characteristic curve. The associations between the factors, and outcome variables were described using the odds ratio and 95% confidence level. The statistical significance of variables was determined at *p*-value ≤0.05.

### Ethical consideration

The ethical review committee of the College of Medicine and Health Sciences, Bahir Dar University reviewed the research protocol, approved the research and gave ethical clearance. The Amhara Regional Health Bureau and East Gojjam Zone Health Department gave supporting letters after being informed about research objectives and procedures by the principal investigator. Verbal informed consent was taken from respective District Health Offices and each TB patient prior to data collection.

## Results

### Socio-demographic characteristics

A total of 348 PTB patients were included in the analysis, where more than half, 209 (60%), 226 (65%), and 205 (59%)) of them were males, under 35 years, and from the rural settings, respectively. Over a third, 129 (37%) of the TB cases had no education, and 188 (54%) of them lived in areas that required traveling for two hours and above for TB services (Table 1).

**Table 1:** Sociodemographic descriptions of PTB patients in East Gojjam zone, Ethiopia, 2018

Variable	Frequency	Percent (%)
Age < 35	226	65.0
≥35	122	35.0
Sex Male	209	60.0
Female	139	40.0
Religion Orthodox	306	88.0
Muslim	42	12.0
Marital status Single	177	51.0
Married	143	41.0
Divorced	28	8.0
Residence Rural	205	59.0
Urban	143	41.0
Education level Unable to read and write	129	37.0
Primary education (1–8)	108	31.0
Secondary (9–12)	76	22.0
College and above	35	10.0
Occupation Farmer	143	41.0
Daily laborer	111	32.0
Driver	42	12.0
Merchant	28	8.0
Employed	24	7.0
Monthly income ≤500 birr	146	42.0
> 500 birr	202	58.0
Family size ≤3	265	76.0
> 3	83	24.0
Distance from facility ≤2 h > 2 h	188 160	54.0 46.0

### Clinical profile and personal behaviors

Most of the respondents, 327 (94%) and 310 (89%), were new PTB cases and had a cough for 14 days and above, respectively. About 202 (58%) were smear-negative PTB cases and 77 (22%) of them had comorbidities. Of 146 smear-positive TB cases, 21 were retreatment cases. More, 150 (43%) PTB cases visited informal services before health facilities (Table 2).

**Table 2:** Clinical profiles and personal behaviors of PTB cases in Ethiopia, 2018

Variable	Frequency	Percent (%)
TB Contact history Yes	136	39.0
No	212	61.0
Cough presence Yes	310	89.0
No	38	11.0
Sputum presence Yes	147	42.2
No	201	57.8
Forms of TB New positive PTB	146	42.0
New negative PTB	202	58.0
Category of TB New PTB	327	94.0
Retreatment cases	21	6.0
Treatment outcome Cured	126	36.2
Completed	204	58.6
Died	6	1.7
Failure	8	2.3
Default	4	1.1
HIV status Positive	77	22.0
Negative	271	78.0
Other comorbidities^a^ Yes	77	22.0
No	271	78.0
Smoking history Yes	87	25.0
No	261	75.0
Chat chewing Yes	91	26.0
No	257	74.0
Alcohol history Yes	108	31.0
No	240	69.0
Knowledge of TB Good	140	40.0
Poor	208	60.0
First action to TB Visit informal facilities	150	43.0
Visit formal facilities	192	57.0
Presence of stigma Yes	139	40.0
No	209	60.0

^a^other commodities include diabetes mellites and hypertension

### TB infectious Pool

The median *TB management time* of smear-positive PTB cases was 82 days (IQR, 56–121 days). Also, the median infectious period after treatment initiation for smear-positive PTB cases was 35 days (IQR, 33–66 days). By adding 35 days, each new smear-positive case contributed to an estimated infectious period of 117 days. Then the 125 TB cases yielded a total of 14, 625 infectious person days annually. Similarly, there were 202 new smear-negative TB cases, where 57% of them were estimated as smear-negative culture positive. The estimated median *TB management time* for this category was 113 days (IQR, 73-155 days). Then, the115 smear-negative culture-positive cases contributed to a total of 12, 995 infectious person-days.

Likewise, the treatment after failure cases had a median *TB management time* of 278 days, IQR:190–306 days. By adding an average infectious period of 56 days after treatment initiation, each failure case contributed to 334 infectious person days and 2672 infectious person-days in total. In this study, the overall average infectious period of new and repeat PTB cases after treatment initiation was 46 days.

Regarding the contribution of undiagnosed TB cases, 51% of 125 smear-positive detected TB cases (64 cases) were not detected in the study area. Also, 51% of 202 smear-negative PTB cases (103 cases) were not detected. Based on the 57% national smear-negative culture-positive proportion, 57% of 103 undetected smear-negative TB cases (59 cases) were expected to be smear-negative culture-positive undetected TB cases. Then, there were a total of 123 (64 + 59) undetected possibly infectious PTB cases. The estimated median TB management time to each undetected PTB case was 365 days that gave to a total of 44,895 infectious person-days. Lastly, the annual TB infectious pool of the study area and the median of total TB infectious period were 78,031 days (Table 3) and 125 days, respectively.

**Table 3:** Estimated median *TB management time*, and TB infectious pool in Ethiopia, 2018

Category of TB cases	Number of PTB cases per year: Number (%)	*TB management time* in days	Infectious period in days	Total annual infectious person-days
New registered PTB cases (*n* = 327)				
Smear-positive	125 (38.2)	82	117	14,625
Smear-negative	202 (61.8)			
Smear-negative culture positive	115 (57.0)	113	113	12,995
Retreatment registered cases (*n* = 21)				
Default	7 (33.3)	197	253	1771
Failure	8 (38.1)	278	334	2672
Relapse	6 (28.6)	142	198	1188
Not registered cases				
Undiagnosed cases	123 (51.0)	365	365	44,895
The total annual infectious pool				78,031

### Factors associated with the TB infectious period

Based on the multivariable logistic regression analysis, being from rural residence, living in areas that require over two hours walk, a form of TB(smear-negative or smear-positive), having knowledge of TB, being a smoker, having comorbidities, taking alcohol, and stigma were statistically significant factors to the magnitude of TB infectious period.

The PTB cases living in rural settings were three times more likely to have a higher infectious period (OR = 2.95, 95%CI = 1.75–5.00) compared to PTB cases from urban settings. Similarly, PTB cases from places that require less or equal to two hours walk were 60% times less likely to have a high infectious period compared to PTB cases living the places that require over two hours walk to get TB services (OR = 0.40, 95%CI = 0.24–0.66). The form of TB, being smear-positive or negative, was statistically associated with the magnitude of TB infectious period, in which smear-positive cases were four times more likely to have a higher infectious period than the counterparts (OR = 4.32, 955CI = 2.51–7.43). Likewise, PTB cases who had an alcohol history were also three times more likely to have a high infectious period (OR = 2.98, 95%CI =1.50–6.00) than the counterparts. Also, the PTB cases who had comorbidities were over two folds to have a high infectious period (OR = 2.65, 95% CI =1.41–5.00) compared to PTB cases who had no comorbidities. Stigma was also found to be statistically significant for the magnitude of TB infectious period. PTB cases who had encountered stigma form the environment were twice more likely to have a high TB infectious period than the counterpart TB cases (OR = 2.01, 95%CI =1.20–3.41) (Table 4).

**Table 4:** Factors associated with TB infectious period in East Gojjam zone, Ethiopia, 2018

Variables		median infectious period		COR (95% CI)	AOR (95%CI)
		High (%)	Low (%)		
Age in years	< 35	106(30.5)	120 (34.5)	0.75 [0.48–1.17]	–
	≥ 35	66 (19.0)	56 (16.0)	1	
Sex	Male	108(31.0)	101 (29.0)	1.25 [0.82–1.93]	–
	Female	64 (18.4)	75 (21.6)	1	
Religion	Orthodox	144(41.4)	162(46.6)	0.44 [0.23–0.88]	–
	Muslim	28 (8.0)	14 (4.0)	1	
Marital status	Single	81 (23.3)	96 (27.5)	0.80 [0.52–1.64]	–
	Married	72 (20.7)	71 (20.4)	0.30 [0.20–1.12]	
	Divorced	19 (5.5)	9 (2.6)	1	
Education level	Unable to read/write	55 (15.8)	74 (21.3)	0.53 [0.41–1.24]	–
	Primary (1–8)	55 (15.8)	53 (15.3)	0.81 [0.42–1.56]	
	Secondary (9–12)	41 (11.8)	35 (10.0)	0.67 [0.34–1.68]	
	College and above	21 (6.0)	14 (4.0)	1	
Residence	Rural	25(36.0)	80 (23.0)	3.20 [1.94–4.73]	2.95 [1.755.00]
	Urban	47 (13.5)	96 (27.5)	1	1
Occupation	Farmer	71 (20.4)	72 (20.7)	0.69 [0.29–1.67]	–
	Daily laborer	53 (15.3)	58 (16.7)	0.66 [0.27–1.62]	
	Driver	19 (5.5)	23 (6.6)	0.68 [0.24–1.91]	
	Merchant	15 (4.3)	13 (3.7)	0.78 [0.25–2.33]	
	Employed	14 (4.0)	10 (2.8)	1	
Monthly income	≤ 500 birr	66 (19.0)	80 (23.0%)	0.75 [0.50–1.15]	–
	> 500 birr	106(30.5)	96 (27.5%)	1	
Family size	≤ 3	131(37.6)	134 (38.5)	1.10 [0.61–1.64]	–
	> 3	41 (11.8)	42 (12.1)	1	
Distance from	≤ 2 h	79 (22.7)	109 (31.3)	0.52 [0.34–0.80]	0.40 [0.240.66]
facility	> 2 h	93 (26.7)	67 (19.3)	1	1
Contact history	Yes	65 (18.7)	71 (20.4)	0.89 [0.58–1.38]	–
	No	107(30.7)	105 (30.2)	1	
Cough presence	Yes	158(45.4)	153 (44.0)	1.70 [0.84–3.42]	–
	No	14 (4.0)	23 (6.6)	1	
Forms of TB	Smear positive PTB	105(30.2)	41 (11.8)	5.16 [3.24–8.21]	4.32 [2.517.43]
	Smear negative PTB	67 (19.2)	135 (38.8)	1	1
First action to	Visit informalfacility	73 (21.0)	77 (22.5)	0.95 [0.62–1.45]	–
TB	Visit formal facility	99 (28.7)	99 (27.8)	1	
Knowledge of	Good	53 (15.3)	87 (25.0)	0.46 [0.30–0.71]	0.36 [0.210.62]
TB	Poor	119(34.2)	89 (25.5)	1	1
Comorbidity	Yes	53 (15.3)	24 (6.9)	2.82 [1.65–4.83]	2.65 [1.415.00]
	No	119(34.2)	152 (43.6)	1	1
Smoking history	Yes	59 (17.0)	28 (8.0)	2.76 [1.65–4.61]	2.43 [1.204.93]
	No	113(32.5)	148 (42.5)	1	
Chat chewing	Yes	46 (13.2)	45 (13.0)	1.10 [0.67–1.72]	–
	No	126(36.2)	131 (37.6)	1	
Alcohol history	Yes	74 (21.2)	34 (9.8)	3.15 [1.95–5.10]	2.98 [1.506.00]
	No	98 (28.2)	142 (40.8)	1	1
Type of facility	PHCU	124(35.6)	128 (36.8)	0.96 [0.61–1.55]	–
	Hospital	48 (13.8)	48 (13.8)	1	
Stigma	Yes	124(35.6)	90 (25.9)	2.47 [1.58–3.85]	2.01 [1.203.41]
	No	48 (13.8)	86 (24.7)	1	1

## Discussion

In this study, we measured the magnitude of the TB infectious pool using a new approach called TB management time. Accordingly, the estimated TB infectious pool of the studied zone was found to be 56,496 infectious person-days. This is a huge figure that indicates the presence of more infectious cases/detected and undetected/and high TB transmission within the community. This implies that there might be poor access to TB services, service delays, poor community awareness, impaired facility readiness, and transportation problem.

The estimated TB infectious pool is lower compared to similar study findings from the West Gojjam zone, which were 325,410 infectious person-days in 2009 [15], and 81,131 infectious person-days in 2014 [14]. The discrepancy with findings could be attributed to differences in sample size, the period of study, and the size of the infectious period used after the commencement of anti-TB treatment. It is true that there were more TB cases (diagnosed and undiagnosed) before five years in Ethiopia compared to the present, which would contribute more to this variation. Since the total infectious period in each TB category is the product of *TB management time* and the number of TB cases, the sample size is vital to determine the magnitude of TB infectious pool. The former studies used a larger sample size in each TB category than the present study. For instance, the study conducted in 2009 was across all the health facilities in West Gojjam Zone, but it is among the sampled facilities in our study area.

The zonal TB infectious period was the sum of the infectious period of each TB category, where undetected TB cases, smear-positive and smear-negative culture-positive PTB cases contributed the highest proportion (Table 3). The same findings were reported by the previous studies from Ethiopia [14, 15]. Possibly, it could be linked to the route of TB transmission/air droplets/in which the most dangerous sources of TB infection are untreated smear-positive TB cases (detected and undetected) [6, 7, 12, 14]. In addition, these three TB categories were larger in the proportion which has a great role in affecting the magnitude of TB infectious pool. The high infectious period among the undetected TB cases (44,895 days) on the other hand, indicates the presence of more undetected infectious TB cases within the community. It shows that more infectious TB cases are not getting TB services. This is a serious issue to increase the size of the TB infectious period and lower the effectiveness of TB control programs. This might be due to limited diagnostic capacity (access and quality), budget scarcity, deprived facility readiness, transportation, and poor community awareness.

The median *TB management time* in each PTB category was relatively larger compared to the previous findings that used *TB management time*. For instance, the median *TB management time* of smear-positive PTB cases was 82 days in this study, which is higher than 45 days [14] and 73 days [15] of former studies. This indicates that there is TB service delay (patient, and/or facility) [35, 36], the TB control program has not covered the community with potential TB suspects, and there is a need either to strictly adhere with or revise the existing TB control strategy. It might be happening due to geographic inaccessibility of TB services [7, 37], poor facility readiness and service quality [12, 24, 25, 38–40], low community awareness [6, 7, 9] infrastructure problems [7, 37, 38], and poor TB program support. Besides the study period, all the above variables might also be reasons for the variations of TB management time between this and the former studies. At present, over a third of health facilities in the study area do not have sputum microscopy services due to the absence of laboratory technicians, and TB diagnostic inputs including reagents, sputum caps, electric power, and water services. Also, only few private clinics have provided TB services, and no private hospitals, which is different from the situation in West Gojjam [14]. All these situations make TB suspects stay undetected in the community for a long period of time.

Due to the absence of GeneXpert and sputum culture services in the study area, we determined the infectious period after the commencement of anti-TB treatment for the sputum smear TB cases through sputum smear conversion time. However, this procedure was less sensitive and unable to identify species. Thus, there might be missing positive cases and considering non-*mycobacterium TB species* as MTB, which then might affect the size of the infectious period [41]. Alternatively, the infectious period after retreatment initiation was estimated based on recent study findings among MDR-TB patients from the Amhara Region, Ethiopia [11, 14, 34]. These make our study differs from the former studies which estimated infectious periods based on study results obtained from other countries [15].

This study tried to identify potential factors associated with the magnitude of TB infectious period in the study area. Based on that, PTB patients originated from rural settings and places far from the health facilities were more likely to have high TB infectious periods compared to their counterparts (Table 4). These findings imply that there is poor access to TB services, inequity, a vicious circle TB infectiousness, high patient/health system delays, poor infrastructures (information access and transportation), low community awareness, and a need to give special attention in TB control strategies in the rural and remote areas where majority of the study area population is leaving. This finding was supported by findings from the previous studies [6, 7, 11, 34] where residence and distance from nearby health facilities were important predictors of poor TB program performance. Besides the previously mentioned gaps, poor health-seeking behavior, cultural influence, family income, education level, and practice of visiting traditional healers might be factors contributing to the discrepancies in the magnitude of TB infectious periods between rural and urban areas [35, 39, 42–45]. For instance, 150(43%) of PTB cases visited informal facilities before visiting the health facilities in the study area (Table 2).

Similarly, TB cases who had comorbidities showed a higher probability to have a high infectious period compared to TB cases who had no comorbidities. This is in agreement with the findings of the former studies [6, 7, 10, 17, 18, 46] where the burden of TB infection is higher among TB cases who had comorbidities such as HIV/AIDS, diabetes mellitus and hypertension. It could be linked to the immune status of TB cases, drug contraindications, poor food intake, psychological stress and poor treatment adherence that might lead to poor improvement and default from anti-TB treatment follow up either by searching for other options or staying at home without taking actions.

Likewise, personal behaviors (smoking and alcohol intake) were predictors to have a longer infectious period (Table 4), which was also reported by the prior studies [6, 7, 22, 34, 47]. The possible explanation for this could be related to the high dropout rate, poor treatment adherence, peer influence, and forgetting to take medication and appointments after being addicted to alcohol and smoking. Also, there might be an interaction between anti-TB drugs and such substances, which might result in acquiring chronic diseases. These conditions might make the situation serious and higher TB infectious period.

In this study, PTB cases who had good knowledge of TB were more likely to have a lower infectious period compared to the respective groups. It is true that if people have better knowledge about TB, they will easily understand suggestive signs and symptoms of TB, and have better health-seeking behavior that results in early TB diagnosis, treatment, and adherence. In addition, people with better knowledge will play a pivotal role in TB prevention by keeping themselves from acquiring TB infection, limiting TB transmission to others, and making awareness creation to the people living around them. All these conditions could lower the degree of infectiousness and the infectious period in the study area.

Equally, the probability of having a high infectious period among PTB cases who got stigma from the community was twice compared to PTB cases with no stigma (Table 4). The previous studies also stated that TB infection and stigma have a direct relationship: as stigma increased, TB transmission will also be increased and vice versa [48, 49]. The probable explanation for this might be linked to the unfavorable decisions made by the TB cases due to the fear of stigma. They may decide not to disclose their status, visit traditional healers, isolate themselves from social events, default from treatment, and develop stress. These conditions might worsen their health status, and increase infectiousness and infectious period. Moreover, this might potentially be a cause for the emergence of MDR-TB strains.

This study tried to overcome the limitations of the former studies by 1). Including private clinics in the study; 2). Estimating infectious TB periods after treatment/retreatment initiation based on practical sputum smear conversion time and recent Ethiopian studies; 3). Considering the contributions of smear-negative undetected TB cases to the TB infectious pool and 4). Identifying potential factors associated with the TB infectious period, are things added in our study compared to the former similar studies. It is the first study that considers all the TB control/patient paths (Fig. 1) including the factors associated with the paths. However, the accurate estimation of the magnitude of the PTB infectious pool would not be easy in the real world [41]. Therefore, we think, this study may provide better information in estimating TB infectious pool and be helpful for PTB control.

Despite maximum efforts made to minimize, this study has some limitations. The recall bias, using a national smear-negative culture-positive proportion to estimate the zonal smear-negative culture-positive TB cases and estimating the infectious period after the retreatment initiation based on literature might slightly affect the magnitude of the TB infectious period. In addition, estimating the infectious period after treatment initiation using sputum microscopy might have a little impact on the size of the TB infectious period. Also, the absence of internationally agreed up cutoff value to dichotomize the outcome variable may have an impact on the explanation of the infectious period.

## Conclusions

The magnitude of the TB infectious pool in East Gojjam zone is lower compared to the previous similar studies. This study is in support of the previous studies on the applicability of *TB management time* in TB control. The primary contributors of the TB infectious pool were undetected, and smear-positive PTB cases, which are indicators for the presence of poor TB case detection in the study area. Residence, knowledge on TB, a form of TB, smoking, alcohol history, distance from the facility, comorbidities, and stigma were statistically significant factors of the TB infectious period. Working on improving personal knowledge, behavior, culture, access, and quality of TB services, and giving a special emphasis on the early detection and treatment of TB and commodities is crucial to improve TB control.

## Data Availability

The datasets generated during and/or analyzed during the current study are available from the corresponding author on reasonable request.

## Abbreviations

AOR: Adjusted Odds ratio
CI: Confidence Interval
COR: Crude Odds ratio
IQR: Inter-Quartile Range
MDR-TB: Multidrug-Resistant Tuberculosis
MTB: *Mycobacterium tuberculosis*
SPSS: Statistical Packages for Social Sciences
TB: Tuberculosis
